# Choosing between Homologous or Heterologous COVID-19 Vaccination Regimens: A Cross-Sectional Study among the General Population in Italy

**DOI:** 10.3390/ijerph19052944

**Published:** 2022-03-03

**Authors:** Marco Clari, Alessandro Godono, Beatrice Albanesi, Elena Casabona, Rosanna Irene Comoretto, Ihab Mansour, Alessio Conti, Valerio Dimonte, Catalina Ciocan

**Affiliations:** 1 Department of Public Health and Pediatrics, University of Torino, 10126 Turin, Italy; marco.clari@unito.it (M.C.); elena.casabona@edu.unito.it (E.C.); rosannairene.comoretto@unito.it (R.I.C.); ihab.mansour@unito.it (I.M.); alessio.conti@unito.it (A.C.); valerio.dimonte@unito.it (V.D.); catalina.ciocan@unito.it (C.C.); 2 Città Della Salute e Della Scienza University Hospital, 10126 Turin, Italy

**Keywords:** COVID-19, vaccines, heterologous, vaccine uptake, safety

## Abstract

A shortage of COVID-19 vaccines and reports of side-effects led several countries to recommend a heterologous regimen for second vaccine doses. This study aimed to describe the reasons behind individuals’ choices of a homologous or a heterologous second vaccination. This cross-sectional study enrolled individuals under 60 who had received a first dose of Vaxzevria and could choose between a homologous or heterologous regimen for their second dose. Quantitative (socio-demographic, clinical characteristics) and qualitative data were collected and analysed through a generalized linear model and thematic analysis, respectively. Of the 1437 individuals included in the analysis, the majority (76.1%) chose a heterologous second dose of the COVID-19 vaccination. More females chose a heterologous vaccination regimen (*p* = 0.003). Younger individuals also tended to choose heterologous vaccination (*p* < 0.001). The main motivation in favour of heterologous vaccination was to follow the Italian Ministry of Health recommendations (*n* = 118; 53.9%). This study showed that most individuals, mainly younger people and females, chose a heterologous dose of COVID-19 vaccination after their first viral vector vaccine. Heterologous vaccinations could be an effective public health measure to control the pandemic as they are a safe and efficient alternative to homologous regimens.

## 1. Introduction

Since the first months of 2020, the coronavirus disease 2019 (COVID-19) pandemic impacted the entire world severely. To date, many countries are still struggling with this global emergency [1].

Initially, non-pharmaceutical interventions, such as social distancing, wearing personal protective equipment [2], quarantine, isolation, and lockdown measures have been at the forefront of outbreak control [3]. Although these measures were quickly strengthened, the outbreak continued to increase, causing severe consequences that negatively affected socio-economic activity [4] and health-related outcomes [5].

Thus, despite all efforts made to limit virus transmission, active immunisation against severe acute respiratory syndrome coronavirus 2 (SARS-CoV-2) by vaccination is currently the cornerstone of global healthcare policies against COVID-19 [6]. To date, five COVID-19 vaccines have been authorized in the European Union after evaluation by the European Medicines Agency (EMA) [7]; two m-RNA vaccines: BNT162b2 (Comirnaty^®^) and mRNA-1273 (Spikevax^®^); two viral vector vaccines: ChAdOx1 nCoV-19 (Vaxzevria^®^) and Ad26.COV2.S (Janssen^®^); and one protein-based vaccine: NVX-CoV2373 (Novavax^®^). Other than with the Janssen vaccine, people need to receive two doses of these vaccines in order to obtain a sustained immunization. A homologous immunization involves the administration of the same vaccine multiple times, while a heterologous immunization involves the administration of different types of vaccines. In the first phase of the COVID-19 vaccination campaign, the administration of a homologous regimen had been proven effective. However, as the pandemic evolved, evidence showed that a heterologous immunization was just as effective as the homologous in terms of immune response [7].

Despite the proven efficacy in terms of reducing infection rates, mortality and symptom severity of both m-RNA and viral vector vaccines [8,9], the actual challenge faced by policymakers is to promote the vaccination campaign in order to achieve optimal immunization coverage. Although, nowadays, general population data indicate mostly positive attitudes towards vaccines, there is still a substantial proportion of individuals who are unsure of their safety and effectiveness, or who outright distrust them. These concerns were exacerbated mainly in the early stages of the vaccination campaigns by the steady growth of misinformation and conspiracy theories. Mistrust and uncertainty also significantly increased following the report of some cases of vaccine-induced thrombosis and thrombocytopenia syndrome (VITTs), mainly involving young women using contraceptive drugs [10].

Worries about VITTs following viral vector vaccines has led several countries to recommend a m-RNA vaccine as the second vaccine dose for adults aged under 60 who had been given a viral vector vaccine for their first dose [11]. Heterologous vaccine regimens have already proved effective in eliciting a good immune response against several pathogens [12].

Unlike other countries, the Italian Medicines Agency (Agenzia Italiana del FArmaco—AIFA) in Italy continued to allow under-60s to get a second dose of Vaxzevria after signing an informed consent form.

So far, no other studies had been conducted to identify factors associated with individuals’ choice of a homologous or a heterologous second dose of the COVID-19 vaccination. Thus, the aim of this study was to describe the reasons underlying individuals’ choices of a homologous or heterologous second dose after an initial dose of the adenoviral COVID-19 vaccine.

## 2. Materials and Methods

This is a cross-sectional study conducted from 19 June to 1 July 2021. Before data collection, participants were informed about the study and provided voluntary consent to participate. Participants could withdraw from the study at any time by asking the researchers to withdraw their data from analysis.

### 2.1. Participants and Data Collection

Participants were individuals who had received a first dose of the Vaxzevria COVID-19 vaccine between 27 March and 8 April 2021. During March and April 2021, Vaxzevria was used to immunize a prioritised category (i.e., university teachers) to enable them to continue to teach in person while avoiding infection clusters [13]. The participants were recruited at the vaccination clinic of the University of Torino, and all the subjects had at least a bachelor’s degree. Following the Italian government resolution of 11 June 2021 [14], individuals under 60 years old could choose whether to receive a heterologous vaccine or a homologous vaccine for their second dose. The study site, located in north-west Italy (i.e., Piedmont), was a choice of convenience. At that time, Piedmont had a cumulative number of 320,094 [15] COVID-19 cases. To be included in the study, individuals had to be aged between 18 and 60 years. The study was approved by the Bio-Ethics Committee of Torino University (Approval No. 0596391).

While waiting to choose which vaccination to receive as the second dose, participants were informed about the study rationale, aims, and anonymity. They were informed that no personal contacts and identifying information would be collected and that all the data would be treated confidentially and used for research purposes only. Moreover, they were informed that they could withdraw from the study at any time and that refusal to participate would not entail any consequences. Participants did not receive any incentive to participate in the study. All the data were stored in a locked closet, accessible only by the research team. The complete database was accessible only by the principal investigator and the corresponding author.

### 2.2. Instruments

Socio-demographic characteristics (gender, age) and relevant clinical characteristics (presence of coagulopathy, cardiopathy, use of hormonal contraceptives, relevant changes in health status in the period between the first and the second dose) of the participants were collected through the medical history established during their screening before vaccination. These variables were selected according to the literature and were validated by a multidisciplinary group of experts.

Moreover, before the immunization, qualitative data were collected through a structured interview responding to the following open-ended question: “Why did you choose to have a homologous/heterologous dose?”. Responses were audio-recorded in a private setting with no one else present in order to guarantee participants’ privacy and to enable them to talk without interruption.

### 2.3. Data Analysis

Characteristics of individuals who chose the homologous versus heterologous regimen were compared using a chi-square test. For categorical variables containing frequencies below five, the *p*-value of the chi-square test was approximated using Monte-Carlo simulations.

A generalized linear model was used to assess the association between the selected independent variables and the choice of receiving a homologous second dose of the COVID-19 vaccine (dichotomous variable). Variables that had statistically significant results in the univariate analysis were included in the generalized linear model. A second generalized linear model including only females was then fitted to include hormonal contraception as an independent variable.

All the analyses were performed using SPSS version 27.0 for Windows (SPSS, Inc., Chicago, IL, USA) and *p*-values < 0.05 were considered statistically significant.

Qualitative data were transcribed verbatim, and two independent researchers (MC, AG) coded them using content analysis [16]. Codes were grouped into key thematic areas until data saturation was reached, understood as the absence of new thematic areas emerging from interviews. It was achieved after 210 interviews, while the last nine interviews were used to confirm the thematic areas already identified. Credibility and dependability were ensured by using an audit trail, verbatim transcription, and member checking with a subsample of participants. Two independent researchers, experts in qualitative research (M.C., B.A.), verified the codes and thematic areas identified, ensuring triangulation. The frequency of different codes was quantified according to the chosen vaccination regimen. The thematic areas arising from this analysis were then compared to identify similarities and differences between groups [17].

## 3. Results

Out of the 1602 individuals aged <60 who had been vaccinated with Vaxzevria as first dose, 1437 were included in the study (response rate 89.7%). A total of 165 individuals were lost to follow-up for the following reasons: refused to participate, became infected with COVID-19 between the first and second dose, or got the second dose in another vaccination clinic for convenience. A total of 292 individuals were asked to respond to the qualitative data collection, with 204 respondents (response rate 69.9%), and 88 who decided not to answer due to privacy reasons. 

Of the 1437 individuals included in the analysis, the majority (76.1%) chose a heterologous COVID-19 vaccination regimen. Participants were mainly males (54.3%) with a mean age of 40.9 (SD = 10.5, 95% CI, 40.4–41.5). All the participants lived in the metropolitan area of Torino and had a middle-high income, ranging from EUR 1500 to about EUR 5000 per month.

Individuals aged >40 years exhibited higher acceptance of the COVID-19 vaccination (52.3%). Out of the 1437 participants, 7.7% (*n* = 111) reported having cardiovascular disease and nine (0.6%) reported coagulopathy. Overall, 7.3% of the women stated that they used hormonal contraception, and the health status of four people changed between the first and second dose.

As reported in Table 1, females were more likely to choose a heterologous vaccination regimen (79.6% vs. 73.2%, *p* = 0.005). In addition, among age bands, younger individuals more frequently chose a heterologous regimen (20–29 years: 83.3%, 30–39 years: 77.7, 40–49 years: 79.2, 50–60 years: 67%, *p* < 0.001). Lastly, more women using hormonal contraception decided not to opt for the homologous vaccination regimen (89.5% vs. 77.7%, *p* = 0.016).

As reported in Table 2, the choice of a heterologous regimen was associated with female gender (OR = 0.684, 95% CI: 0.533–0.877) and a younger age (20–29 years: OR = 0.399, 95% CI: 0.274–0.582). Furthermore, in the women subgroup, younger women had a higher probability of choosing a heterologous regimen (20–29 years: OR = 0.251, 95% CI: 0.126–0.500). No association was found with the use of hormonal contraception (Table 3). Both models had good fit indexes. 

### Motivation Expressed by Participants

The main motivation stated by participants in favour of heterologous vaccination was to follow recommendations of the Italian Ministry of Health (*n* = 118, 53.9%), which were adopted mainly by younger subjects (mean age 40 years). Only a small proportion (14 out of 219; 6.3%) asked for a consultation with their General Practitioner about which regimen they should choose. Four of them were recommended to choose a homologous regimen (Table 4). Particularly, females and younger subjects (43 years, SD 12.1) were recommended to choose an mRNA vaccine, whereas older participants were recommended homologous vaccination (51 years, SD 8.5).

The rest of the individuals stated specific personal reasons in favour of choosing either homologous or heterologous vaccination against COVID-19. Among those who opted for the second dose of Vaxzevria (44 years, SD 9.6), 30 respondents preferred continuity of care, while 23 respondents decided to continue with Vaxzevria because they perceived it as safer than Spikevax. Two respondents stated concerns about the mix-and-match vaccination regimen. Finally, 3 of the 58 individuals chose the Vaxzevria second dose because they had not experienced side-effects after the first dose.

Among the 29 respondents who chose the heterologous regimen, seven had safety concerns about receiving a second dose of a COVID-19 viral vector vaccine. In fact, concerns about this vaccine were expressed mainly by females (4 out of 7) and younger individuals (43, SD 11.3). Overall, 13 out of the 29 individuals were in favour of the mix-and-match regimen, as they were informed that it was more effective than the homologous regimen. Finally, four respondents stated that their choice was influenced by side-effects experienced following the first dose with Vaxzevria.

## 4. Discussion

The main objective of our study was to identify the factors underlying choices of a homologous regimen or a heterologous second dose after a first dose of a viral vector COVID-19 vaccine.

More than 75% of the study sample chose to complete the vaccination course with an mRNA vaccine, thus preferring a heterologous regimen to a homologous one. The main finding of the statistical analyses was that being women, being younger, and taking contraceptive drugs were factors associated with the choice of a heterologous regimen. This statistically significant difference between genders is relevant and worth discussing. Recent publications have already investigated the differences between men and women in terms of SARS-CoV-2 vaccines safety and efficacy. A recent systematic review and meta-analysis by Zhu et al. [18] showed no significant gender differences in terms of efficacy of the COVID-19 vaccines, especially in younger populations. Conversely, in terms of safety, a recent study pointed out that more females report adverse events following COVID-19 vaccinations compared to males, while males are more likely to have serious adverse events, hospitalizations, and deaths [19].

Gender also seems to be an important factor in promoting COVID-19 vaccines. Indeed, a study by Lazarus et al. [20] assessed the association of demographic factors with vaccine acceptance from a random sample of 13,426 participants selected from 19 high-COVID-19 burden countries. Their results showed high heterogeneity in terms of gender differences at the national level, and this variance could be probably attributed to complex socio-environmental, psychological, and cultural influences.

Furthermore, our results highlighted that women aged <60 years are more likely to choose a heterologous vaccine regimen than men. This finding is probably due to the media dissemination of exaggerated information about thromboembolic adverse events caused by viral vector vaccines, especially in females. Several articles [21,22] have already studied the role of mass-media communications during the COVID-19 outbreak with regard to the vaccination campaign. Scientific literature substantially agrees that there are inaccuracies, errors of both fact and logic, and a clear lack of comprehensive information, concluding that media often misrepresent clinical evidence and its bearing. Therefore, it is particularly important to avoid the transmission of inaccurate information to prevent misinterpretation and wrong decisions about getting vaccinated.

Another relevant consideration from our analysis is that younger participants seem to be more likely to choose the heterologous regimen rather than the homologous. Historically, along with endorsing different reasons for vaccinating, age cohorts exhibit different rates of vaccine confidence and uptake. Older age is frequently reported as a good predictor of vaccination intention and it has been recently identified as a factor in greater adherence to COVID-19 public health guidelines [23]. A possible explanation of this apparent discrepancy with current scientific literature could be that participants aged between 50 and 59 years old chose the homologous regimen because of their proximity to the age-limit of 60 identified by the Italian Health Ministry.

Regarding the other health information obtained by standardized questionnaires, there is a clear trend towards an under-reporting of comorbidities and prescribed therapies. Only a small proportion of the subjects reported having cardiovascular diseases (CVDs) or taking contraceptive drugs, while the prevalence of CVDs is almost 40% between ages 40 and 59 [24] and the prevalence of contraceptive drugs in Italy is 16.2% [25]. Such under-reporting is also a known issue in other contexts [26] that takes on relevance before administering a vaccine. A possible reason underlying this phenomenon could be patients’ perception that reporting comorbidities is not relevant for vaccination. Moreover, fear of being excluded from vaccination can discourage patients from being completely transparent [27]. Therefore, during the anamnestic interview, the vaccinating physician should focus on pathological conditions and treatments.

Trust in public health recommendations is a key psychological factor behind vaccine acceptance [28]. This result also emerged from the analysis of participants’ motivations, in which following government recommendations was one of the main reasons for choosing a heterologous vaccination regimen. Furthermore, personal reasons, such as vaccine-related safety and disease issues, emerged among the motivations for choices of both vaccination regimens. These factors are reported as a major concern among the general population, playing a significant role in adherence to vaccination programmes [29]. Lastly, another relevant insight emerging from the analysis of motivations is that only a small proportion of the participants requested a consultation with their General Practitioner (GP). In our opinion, this is a major issue since GPs’ advice could potentially reach many people.

### Strengths and Limitations

This study has some strengths and limitations that need to be stated. The main strength is the novelty of the work: to the best of our knowledge, no other study has covered this topic. The use of a quantitative-qualitative methodology allowed a more complete description of the phenomenon. Moreover, even though the participants are not representative of the whole Italian population, the sample size is sufficient to allow some reflections on the general population. Furthermore, all the participants had a university level degree, which could have influenced the level of compliance to the study.

Our study also had some limitations. The main limitation is the number of variables collected. Nevertheless, we consider that the variables in the study were those most relevant for the understanding of the phenomenon. Moreover, our study is monocentric and cross-sectional. A further limitation is that the qualitative results may not be extendable to other contexts. During the COVID-19 pandemic, emotional issues could have influenced the responses, leading to recall bias. However, the credibility and generalizability of our findings were increased using quantitative and qualitative data collected from a large sample.

## 5. Conclusions

This study showed that most participants chose a heterologous regimen, particularly younger individuals, and females. Uncertainty about the safety of viral vector vaccines probably led to participants’ choice of a heterologous vaccine regimen, but the main reason behind choice of the heterologous regimen was to follow the Italian Ministry of Health recommendations.

Heterologous vaccinations could be an effective public health measure to control the pandemic as they are less time-related, and they are a safe and efficient alternative to homologous regimens. In a time of scarce resources, this could be a solution to extend the vaccination to a wider population, independent of the vaccine supply chain. Our findings can also guide policymakers to improve adherence to heterologous vaccination. In particular, more targeted and effective interventions should be promoted among people at risk of hesitancy. More informative campaigns on the use of mix-and-match vaccination regimens should be realized through a more open dialogue with citizens. Further studies are needed to understand whether our findings can be extended during the unrolling of the vaccination campaign.

## Figures and Tables

**Table 1:** Participants according to the vaccination regimen chosen.

Characteristics			*p*-Value
	Homologous Vaccination (*n* = 343; 23.9%)	Heterologous Vaccination (*n* = 1094; 76.1%)	
Sex *n* (%)			
Male	209 (60.9)	571 (52.2)	0.005
Female	134 (39.1)	523 (47.8)	
Age *n* (%)			
20–29	47 (13.7)	235 (21.5)	<0.001
30–39	90 (26.2)	313 (28.6)	
40–49	77 (22.4)	284 (26.0)	
50 –60	129 (37.6)	262 (23.9)	
Coagulopathy *n* (%)			
Yes	2 (0.6)	7 (0.6)	1.000
No	341 (99.4)	1087 (99.4)	
Cardiovascular disease *n* (%)			
Yes	34 (9.9)	77 (7.0)	0.083
No	308 (90.1)	1016 (93.0)	
Hormonal contraception *n* (%)			
Yes	6 (1.7)	51 (4.7)	
No	337 (98.3)	1043 (95.3)	0.016
Change in health status *n* (%)			
Yes *n* (%)	2 (0.6)	2 (0.2)	0.243
No *n* (%)	341 (99.4)	1092 (99.8)	

**Table 2:** Vaccination regimen chosen by sex and age (all participants): generalized linear model.

	OR	95% CI		*p*-Value
(Intercept)	0.587	0.463	0.744	<0.001
Sex (Female)	0.684	0.533	0.877	0.003
Age				
20–29	0.399	0.274	0.582	<0.001
30–39	0.572	0.417	0.785	<0.001
40–49	0.546	0.392	0.761	<0.001
50–60	§			

Abbreviation: OR: Odds Ratio, CI: Confidence Interval, §: Reference.

**Table 3:** Vaccination regimen chosen by age and hormonal contraception (women): generalized linear model.

	OR	95% CI		*p*-Value
(Intercept)	0.288	0.113	0.733	0.009
Age				
20–29	0.251	0.126	0.500	<0.001
30–39	0.459	0.277	0.762	0.003
40–49	0.589	0.363	0.956	0.032
50–60	§			
Hormonal contraception (Yes)	1.571	0.638	3.870	0.326

Abbreviation: OR: Odds Ratio, CI: Confidence Interval, §: Reference.

**Table 4:** Participants’ motivation according to the chosen vaccination regimen.

Motivation	Total Participants *n* (%)	Females *n* (%)	Males *n* (%)	Mean Age (SD)
Health Ministry Recommendation	118 (53.9)	54 (24.7)	64 (29.2)	40 (10)
Medical consultation	14 (6.4)	7 (3.2)	7 (3.2)	45 (11.6)
Homologous	4 (1.8)	1 (0.4)	3 (1.4)	51 (8.5)
Heterologous	10 (4.7)	6 (2.7)	4 (1.8)	43 (12.1)
Personal choices in favour of Vaxzevria	58 (26.5)	24 (11)	34 (15.5)	44 (9.6)
Care continuity	30 (13.7)	16 (7.3)	14 (6.3)	43 (9)
Belief (convinced opinion)	23 (10.5)	7 (3.1)	16 (7.3)	46 (10)
Fear of heterologous regimen	2 (0.9)	1 (0.4)	1 (0.4)	47 (1.4)
No side-effects after first dose	3 (1.4)	0	3 (1.4)	35 (12.4)
Personal choices in favour of Spikevax	29 (13.2)	12 (5.4)	17 (7.8)	44 (11.6)
Belief (convinced opinion)	13 (5.9)	3 (1.4)	10 (4.7)	45 (12.5)
Fear or conflicting opinion on Vaxzevria	7 (3.2)	4 (1.8)	3 (1.3)	43 (11.3)
Other reasons (i.e., advice of friends)	5 (2.2)	2 (0.9)	3 (1.4)	49 (13.1)
Health reasons	4 (1.8)	3 (1.4)	1 (0.4)	39 (7.8)
Total	219	97	122	42 (10)

## Data Availability

The data is not available to the public without the consent of the PI Marco Clari.
